# The impact of contact patterns on epidemic dynamics

**DOI:** 10.1371/journal.pone.0173411

**Published:** 2017-03-14

**Authors:** Qiuju Yin, Tianyu Shi, Chao Dong, Zhijun Yan

**Affiliations:** 1 School of Management and Economics, Beijing Institute of Technology, Beijing, China; 2 Sustainable Development Research Institute for Economy and Society of Beijing, Beijing, China; Universidad de Zaragoza, SPAIN

## Abstract

In social networks, individuals have relationships with their neighbor nodes (acquaintance contacts) and also randomly contact other nodes without direct links (stranger contacts). However, these two types of contact patterns are rarely considered together. In this paper, we propose a modified SIS (Susceptible-Infected-Susceptible) model in which a node not only contacts neighbor nodes but also randomly contacts other nodes in the network. We implement the model on a scale-free network and study the influence of different types of contact patterns on epidemic dynamics as well as three possible strategies people adopt when disease outbreaks. The results show that a greater preference for acquaintance contacts makes a disease outbreak less likely. Moreover, the best protective strategy to control the disease is to adjust both the contact number and the contact pattern. In addition, the epidemic is more likely to be controlled when individuals take more information into consideration.

## Introduction

Large-scale outbreaks of infectious diseases result in high mortality and morbidity and also create huge economic burdens for society. Therefore, research on the mechanism of contagion and measures to prevent spread are always of interest. Many infectious diseases spread through physical contact, which follows different patterns for different people. The contact patterns of the population determine who is most at risk for infection and greatly affect how the epidemic spreads [1]. Therefore, to understand the disease spreading process and propose effective control strategies, it is necessary to incorporate contact patterns into infectious disease transmission models.

Previous studies analyzing disease spreading mostly considers two main mixing patterns of the population. The first is homogeneous mixing, and the second is based on network structure; corresponding to the two mixing patterns, mathematical models and complex network theory are the two most popular tools for infectious diseases modeling [2, 3]. Mathematical models have long been applied to study the spread of infectious diseases and are still widely used [4, 5]. A traditional mathematical model is expressed in the form of differential equations and is based on mean-field theory. With the emergence and development of complex network theory, scholars used complex networks to describe the contact networks of the population and to study the disease transmission process and control strategies on the network structure [6–8]. In the contact network, nodes represent individuals, and links (or edges) correspond to the relationships among people. Viruses can spread from one node to the other node through the links (edges) between them.

However, most existing studies are based on some assumptions that differ from the real world. The traditional mathematical disease models assume that the population is fully mixed such that each individual has an equal chance of contacting any other member of the population [9, 10]. Due to the complexity of the contact patterns of people, this simple assumption cannot completely capture the real spatial structure and interactions of a population. The complex network-based models make an assumption that an individual can contact their social or spatial neighbors based on the network structure [7, 11, 12]. Small-world, random, scale-free and regular networks are the most commonly used network structure models. Although these network models help to analyze and imitate the complex spreading process of infectious diseases, it is not all-inclusive. People in the real world always communicate with others in different ways. At the same time, they not only contact their friends but also may inevitably contact strangers, which is referred to here as temporary contact. Temporary contact is transient and random with respect to time and place, which may affect the spread of disease. In other words, the contact network is not an absolute static network or fully mixed, but rather a network that adds dynamic contacts to a basic static network.

In response to a disease outbreak, people may take protective measures to reduce their infection risk, including receiving vaccination, reducing contact frequency, avoiding contact with the infected, and taking everyday precautions (such as washing hands, wearing masks, etc.) [8, 13–21]. Reducing contact intensity is an effective and cost-saving measure that people often adopt voluntarily. V.S. Del et al. applied a mathematical model to study the effects of reducing daily contact activity rate in a smallpox attack and found that even gradual and mild behavior changes can have a dramatic impact on slowing the spread of the epidemic [22]. However, previous studies ignore contacts with strangers and always model the effect of contact adjustment using a change in parameters (infection rate or susceptibility rate). Focusing on the contact adjustment, we divide infection rate into the transmission probability per contact and the contact number per time. The transmission probability per contact is constant, while the contact number changes with time. In addition, most studies assume that behavioral responses are based on disease-related information. In this study, we assume that people adjust their contact number according to the three following types of information: contact information, local information and global information. We propose three strategies that people may adopt in response to a disease outbreak and investigate their effect on disease spread while considering contact with strangers.

The rest of this paper is arranged as follows. In Section 2, the proposed model is briefly described, and the definitions of two contact patterns and three disease response strategies are presented. We investigate the influence of different contact patterns on disease spread and provide the simulation results in Section 3. Section 4 gives the conclusions.

## Model

The susceptible-infected-susceptible (SIS), susceptible-infected-recovered (SIR), and susceptible-infected (SI) models are the classical models for studying the spread of disease [11, 12, 23, 24]. In this study, we adopt the susceptible-infected-susceptible (SIS) model to study the effect of different contact patterns on transmission dynamics of disease. In the SIS model, a susceptible (S) node will be infected at a rate of β after contact with an infected neighbor. At the same time, an infected (I) node will return to the susceptible state at a rate of γ, yielding an effective transmission ratio *λ* = *β*/*γ*. Without losing generality, we can set *γ* = 1 [9].

Considering that people accumulate many friends over time, and the number of friends varies among individuals, scale-free networks were adopted to describe the scale-free characteristic of the social contact network in the real world [24]. The classical disease transmission on scale-free networks assumes that each node can contact all their surrounding neighbors at each time point. Pastor-Satorras and Vespignani studied the SIS model on scale-free networks using this assumption, and simulation and analytic results found that the SIS model with a scale-free network does not show threshold behavior; in other words, even diseases with very low infectivity have the potential to cause epidemics [12]. Rui Yang studied the SIR model using scale-free networks with identical infectivity and assumed that each node could only generate identical and constant contacts with neighbors; simulation and analytic results showed a threshold behavior in this model that was independent of network structure and inversely proportional to the number of contacts [24].

In this study, we consider new contact patterns that differ from previous studies and propose three further strategies. We provide a detailed introduction in the following three parts.

### Contact patterns

Contact patterns determine individuals’ interactions with others. They further determine disease evolution from a global perspective as well as infection risk from an individual perspective. Most previous models of epidemic spreading based on complex networks that assume viruses can only spread through links (representing social relationships) are not perfect. In this study, we classify contact patterns as acquaintance contacts and stranger contacts according to whether the contact occurs among the nodes with social relationships. We define contacts through the existing social relationship (network edges) as acquaintance contacts, while temporary and random contacts among strangers are defined as stranger contacts. Obviously, these two types of contact patterns have essential differences. Acquaintance contacts occur in a relatively local, small and fixed set of nodes (referred to as circle of friends). However, stranger contacts may occur between any two nodes in the entire contact network. From a long-term perspective, acquaintance contacts are repeated and have a high contact frequency with the same individual. By contrast, stranger contacts always represents new contacts with different people, and the inevitable physical contacts between strangers vary greatly with time (there is a low probability of meeting the same stranger twice in a relatively large population). Social relationships are different from social contacts. In our study, the social network is static during the spread of the disease (a relatively short time), which rules out the possibility of new social ties forming, and we add stranger contacts to the static social network to obtain a dynamic contact network.

We assume that each node makes *A*_*0*_ contacts with other nodes at each time point (without considering self-protective measures), where *A*_*0*_ is the same for every node [11, 24]. A node is allowed to contact with the same node multiple times. Individuals can simultaneously contact friends and strangers. We define the proportion of acquaintance contacts as μ and the proportion of stranger contacts as 1-*μ*. We define the acquaintance contacts number as *A*^*A*^ *= A*_*0*_μ and the stranger contacts number as *A*^*S*^ *= A*_*0*_ (*1-μ*). We study the effect of μ on two main properties of epidemic dynamics, including the threshold and the final size of the spread of epidemic.

### Adjustment of contact number according to disease information

When a disease outbreak occurs, people adjust their contact behavior to protect themselves from infection. In the era of information, people adjust their contact behavior based on various information obtained from different channels, including social networks, newspapers, and public health authorities. Information about disease can inspire protective awareness of people. In a prior publication [25], the author classified information into prevalence-based information or belief-based information according to the type of information and classified the information into global information or local information according to the source of information. Different articles use different sources and types of information to study the effect of information-driven behavior changing on the epidemic dynamics.

In this study, we consider the following three forms of information: contact information, local information and global information [26, 27], which together can better capture the diversity and complexity of real information. Local information comes from social or spatial neighbors, and it refers to the infection proportions of the surrounding neighbors within three degrees of separation, which differs from previous studies. Most previous studies assume that local information refers to the infection proportion of direct neighbors. However, based on the Three Degrees of Influence Rule proposed by N.A. Christakis et al. [28], people’s behaviors are influenced not only by their direct friends but also by their friends’ friends and their friends’ friends’ friends. In addition, due to the dissemination of information in social networks, individuals can acquire infection information about neighbors’ neighbors or neighbors’ neighbors’ neighbors and then adopt protective behavior before an infection occurs in a direct friend. Thus, for local information, the influence of infection proportions within three degrees of separation is considered. Global information is publicly available to everyone and refers to the infection proportion of the whole population. Local information and global information are prevalence-based information. Contact information is belief-based information and refers to the node degree, which implies that the greater degree a node possesses, the higher risk a node perceives.

We introduce 1/*k*_*i*_ to characterize contact information, where *k*_*i*_ represents the degree of node *i*. The bigger the degree of the node *i* has, the higher risk of infection the node *i* perceives. Local information refers to infection proportions of the individual’s neighbors within three degrees of separation. $I_{i}^{1}(t)/k_{i}^{1}$, $I_{i}^{2}(t)/k_{i}^{2}$, and $I_{i}^{3}(t)/k_{i}^{3}$ represent the infection proportion of node *i*’s direct neighbors, infection proportion of node *i*’s neighbors’ neighbors, and infection proportion of node *i*’s neighbors’ neighbors’ neighbors at time *t*, whereas $k_{i}^{1},k_{i}^{2}\;\text{and}\;k_{i}^{3}$ represent the number of node *i*’s direct neighbors (equal to the degree of node *i*), the number of node *i*’s neighbors’ neighbors, and the number of node *i*’s neighbors’ neighbors’ neighbors, and $I_{i}^{1}(t),I_{i}^{2}(t)\;\text{and}\;I_{i}^{3}(t)$ represent the infection number of node *i*’s direct neighbors, the infection number of node *i*’s neighbors’ neighbors, and the infection number of node *i*’s neighbors’ neighbors’ neighbors at time *t*. Given that social influence decays with social distance among individuals (the father the social distance, the less the social influence), our model introduces δ as the attenuation coefficient and defines $L_{i}=\frac{I_{i}^{1}(t)/k_{i}^{1}+(1-\delta )I_{i}^{2}(t)/k_{i}^{2}+{\left(1-\delta \right)}^{2}I_{i}^{3}(t)/k_{i}^{3}}{1+(1-\delta )+{\left(1-\delta \right)}^{2}}$ as the sum local information with three degrees of separation. As shown in the above equation, with increasing attenuation coefficient δ, the social influence of neighbors at farther distance will be smaller. Particularly, δ = 1 means that only direct neighbors can exert influence on the contact behavior of an individual; δ = 0 means that all neighbors have the same influence on contact behavior of an individual, regardless of social distance. We introduce *G*_*i*_ = *I*^*g*^(*t*)/*N* to characterize global information received by node *i* at time *t*, *I*^*g*^(*t*) refers to the number of infected individuals in the entire network and N is the network size.

The initial contact number is *A*_*0*_, and individuals will take the three types of information described above into account to adjust the contact number. Different information exerts different influences on the contact behavior of people. Referring to [26, 27], the equation for the contact number of node *i* at time *t* is given as follows:

$$A_{i}(t)=A_{{}_{0}}(1-bL_{i})(1-cG_{i})/k_{i}^{a}$$ (1)

Where *a*, *b*, and *c* are the influencing factors of contact information, local information and global information, respectively, and *a* ≥ 0, 0 ≤ *b* ≤ 1, 0 ≤ *c* ≤ 1. Considering that the node will have acquaintance contacts and stranger contacts, we have $A_{i}(t)=A_{i}^{A}(t)+A_{i}^{S}(t)$.

### Protective strategies

The contact behavior of an individual consists of two important properties: contact number and contact patterns. From the above section, we can determine the contact number of each node at each time according to three types of information. However, when faced with the risk of disease, individuals may show different responses. Some people do not react to the spread of disease and take no protective measures. Some think that reducing contact number is important (exposure to acquaintances and contact with strangers has the same risk of infection). Others are more sensitive to outside risk and think it is safer to have contact with familiar people than with strangers, so they reduce travel and avoid crowded places. Thus, people may use one of the following three strategies: doing nothing (strategy 1), changing only contact number (strategy 2), or adjusting both contact number and contact patterns (strategy 3).

Strategy 1 indicates that people maintain normal contact behavior as if no disease was present. Namely, at any given time, people maintain the same contact number and have an unchanged ratio of acquaintance contacts to stranger contacts. Thus, we have:

$$A_{i}(t)=A_{0},\;A_{i}^{A}(t)=A_{0}\mu ,\;A_{i}^{S}(t)=A_{0}(1-\mu ).$$

Strategy 2 means that people adjust their contact number according to the disease information, but the proportion of acquaintance contacts and stranger contacts remains unchanged. In this situation, the contact number of node *i* at time *t* can be given as Eq (1), while

$$A_{i}(t)=A_{{}_{0}}(1-bL_{i})(1-cG_{i})/k_{i}^{a}\;A_{i}^{A}(t)=A_{i}(t)\mu \;\text{and}\;A_{i}^{S}(t)=A_{i}(t)(1-\mu ).$$

In strategy 3, individuals not only reduce their contact number according to the disease information (described in Eq (1)) but also change the ratio of acquaintance contacts to stranger contacts (reduce their stranger contacts first and then change their acquaintance contacts). Therefore, the acquaintance contacts number is defined as follows:

$$A_{i}^{A}(t)=\left\{ \begin{array}{l} A_{0}\mu \;when\;A_{i}(t)\geq A_{0}\mu \\ A_{i}(t)\;when\;A_{i}(t)\leq A_{0}\mu \end{array}\right.$$ (2)

Then, according to $A_{i}(t)=A_{{}_{0}}(1-bL_{i})(1-cG_{i})/k_{i}^{a};\;A_{i}(t)=A_{i}^{A}(t)+A_{i}^{S}(t)$, the stranger contacts number is:

$$A_{i}^{S}(t)=\left\{ \begin{array}{l} A_{i}(t)-A_{0}\mu \;when\;A_{i}(t)\geq A_{0}\mu \\ 0\;when\;A_{i}(t)\leq A_{0}\mu \end{array}\right.$$ (3)

Namely, when individuals perceive some but not very serious risk of infection, they will reduce some stranger contacts (reduce travel, going out and avoid public or crowed places) but keep normal contact with their circle of friends; when they perceive a high risk of infection, they will stay home (only contact their family members and eliminate stranger contacts), avoid public places and stay home from school or work (avoid strangers and some friends).

## Simulations and results

**Fig 1:**
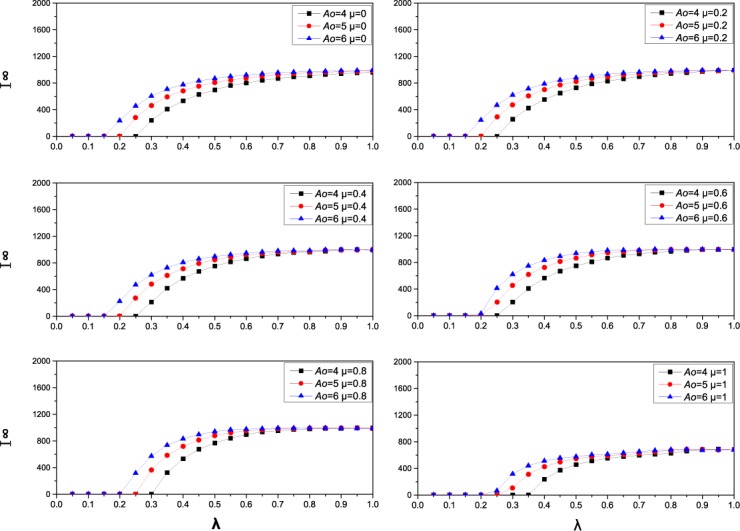
The effect of contact frequency *A*_*0*_ on the prevalence and threshold of epidemic. In each of the 6 panels, I^∞^(the final epidemic prevalence) as a function of the effective spreading rate λ on a scale-free network of 2000 nodes with degree distribution *p*(*k*) ∼ *k*^−2.35^. In each subpanel, there are three color lines, the black, red and blue curves represent contact number *A*_*0*_ = 4,5,6, respectively.

**Fig 2:**
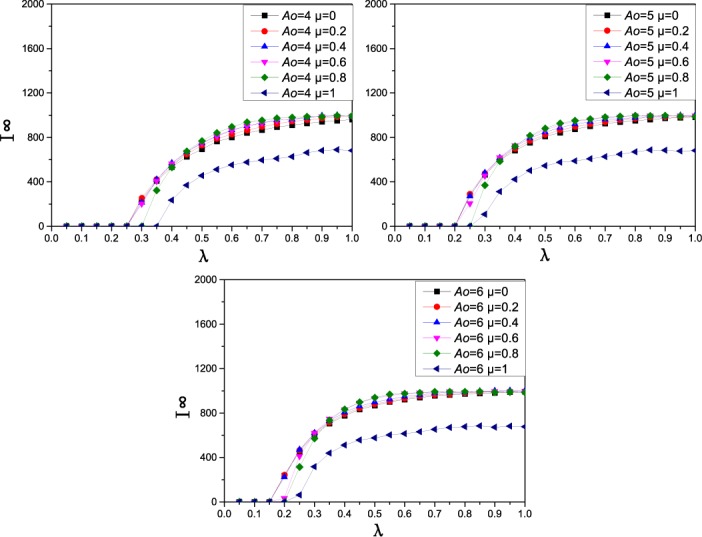
The effect of acquaintance contacts ratio *μ* on the prevalence and threshold of epidemic. In each of the 3 panels, I^∞^(the final epidemic prevalence) as a function of the effective spreading rate *λ* on a scale-free network of 2000 nodes with degree distribution *p*(*k*) ∼ *k*^−2.35^. In each subpanel, there are 6 color lines, that represent different acquaintance contacts ratios *μ* = 0, 0.2, 0.4, 0.6, 0.8, and 1.

**Fig 3:**
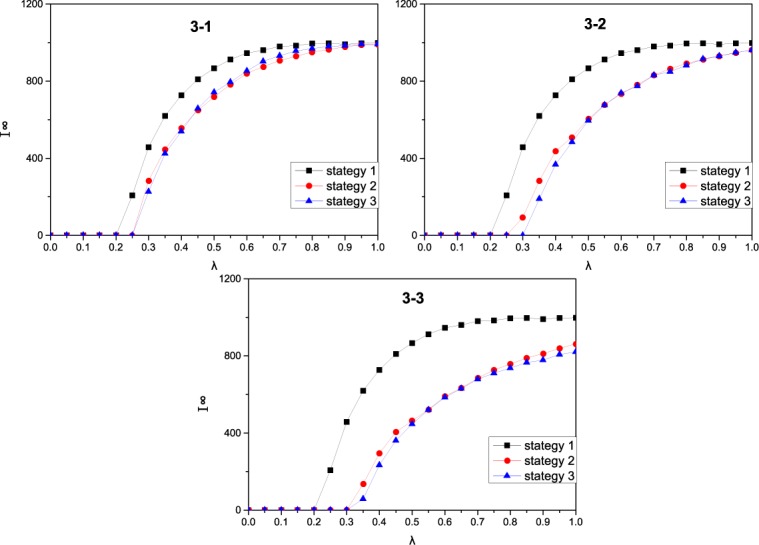
The effect of three strategies on the epidemic dynamics. In each of the 3 panels, I^∞^(the final epidemic prevalence) as a function of the effective spreading rate λ on a scale-free network of 2000 nodes with degree distribution *p*(*k*) ∼ *k*^−2.35^. In each subpanel, there are three color lines: the black, red and blue line, represent strategy 1 without any contact behavior adjustment, strategy 2 (adjustment of contact number) and strategy 3 (adjustment of both contact number and contact patterns), respectively. The other parameters are set to: *A*_0_ = 5,*μ* = 0.6,*a* = 0.2,*b* = 0.2,*c* = 0.2,*δ* = 0.5 in the panel 3–1, *A*_0_ = 5,*μ* = 0.6,*a* = 0.5,*b* = 0.2,*c* = 0.2,*δ* = 0.5 in the panel 3–2, *A*_0_ = 5,*μ* = 0.6,*a* = 1,*b* = 0.2,*c* = 0.2,*δ* = 0.5 in the panel 3–3.

**Fig 4:**
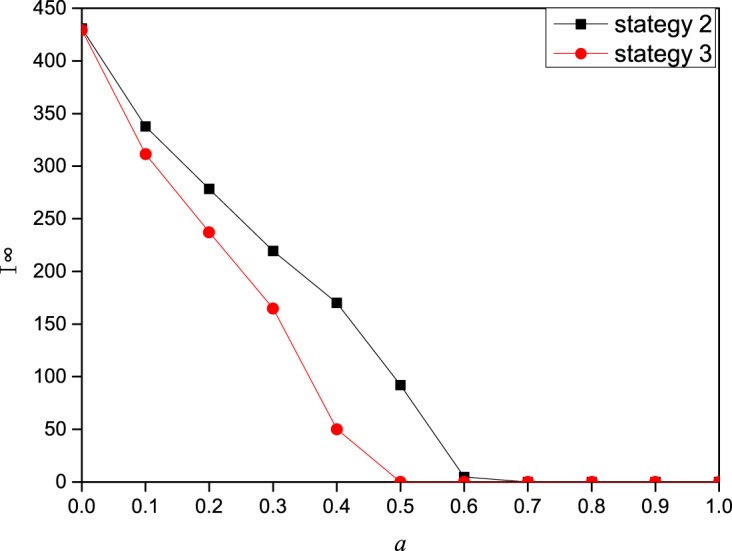
The effect of the contact information influencing factor *a* on the epidemic transmission dynamics. In this figure, I^∞^ (the final epidemic prevalence) as a function of the contact information influencing factor *a* on a scale-free network of 2000 nodes with degree distribution *p*(*k*) ∼ *k*^−2.35^. The other parameters are set to *A*_0_ = 5,*β* = 0.3,*γ* = 1,*μ* = 0.6,*b* = 0.2,*c* = 0.2,*δ* = 0.5.

**Fig 5:**
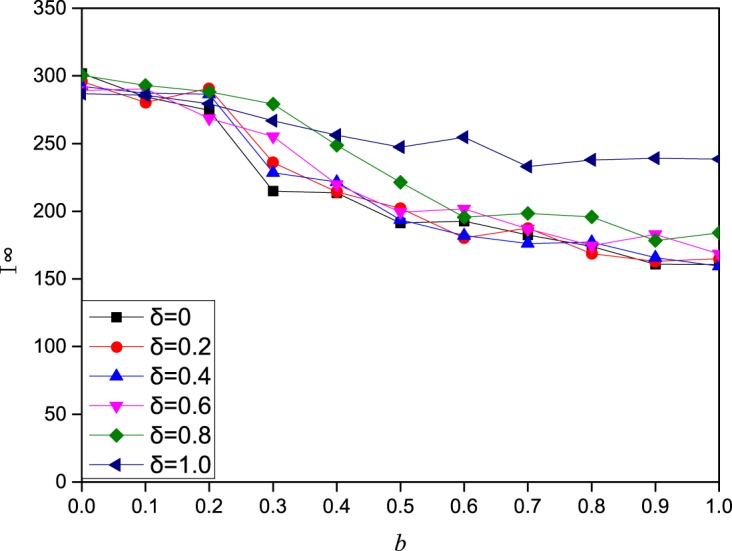
The effect of the local information influencing factor *b* on the epidemic spreading dynamics under strategy 2. In this figure, I^∞^(final prevalence) as a function of the local information influencing factor *b* under strategy 2 on a scale-free network of 2000 nodes with degree distribution *p*(*k*) ∼ *k*^−2.35^. The other parameters are set to *A*_0_ = 5,*β* = 0.3,*γ* = 1, *μ* = 0.6,*a* = 0.2,*c* = 0.2,*δ* = 0.5.

**Fig 6:**
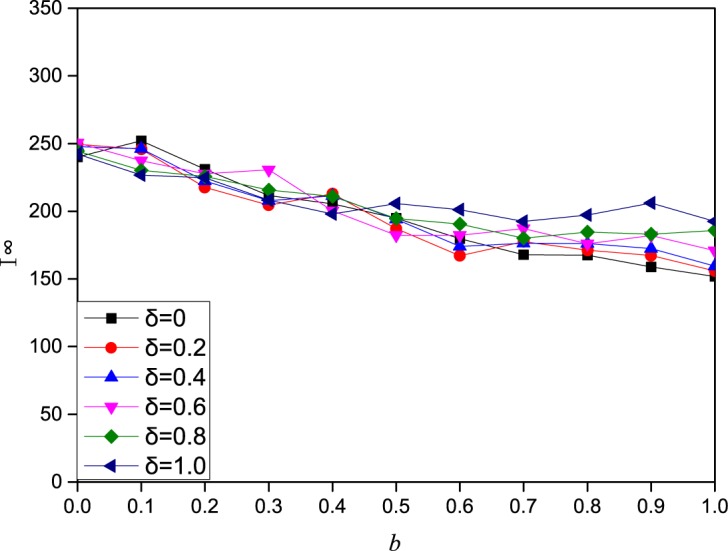
The effect of the local information influencing factor of on the epidemic spreading dynamics under strategy 3. In this figure, I^∞^ (final prevalence) as a function of the local information influencing factor *b* under strategy 3 on a scale-free network of 2000 nodes with degree distribution *p*(*k*) ∼ *k*^−2.35^. The other parameters are set to *A*_0_ = 5,*β* = 0.3,*γ* = 1, *μ* = 0.6,*a* = 0.2,*c* = 0.2,*δ* = 0.5.

**Fig 7:**
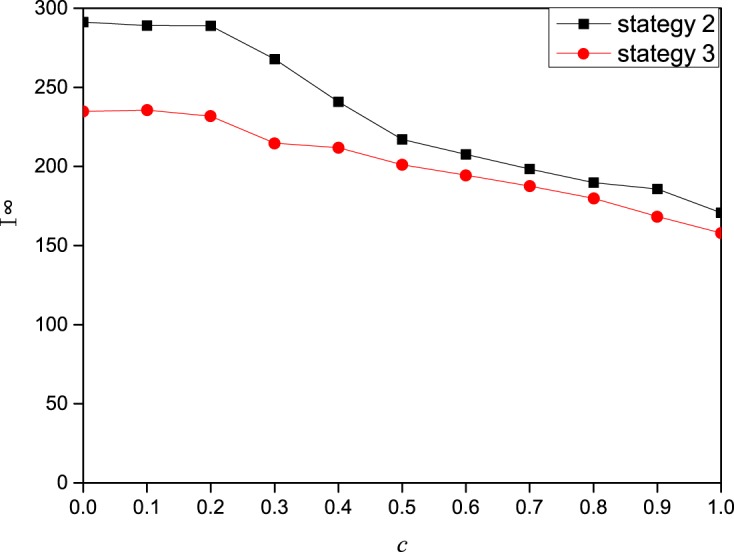
The effect of the global information influencing factor *c* on the epidemic spreading dynamics. In this figure, I^∞^ (the final epidemic prevalence) as a function of the global information influencing factor *c* on a scale-free network of 2000 nodes with degree distribution *p*(*k*) ∼ *k*^−2.35^. The other parameters are set to *A*_0_ = 5,*β* = 0.3,*γ* = 1, *μ* = 0.6,*a* = 0.2,*b* = 0.2,*δ* = 0.5.

Because it is difficult to mathematically describe the disease transmission dynamics of populations with complex structure, we used simulation to study the effect of different types of contact patterns and protective strategies on epidemic dynamics. First, the disease spreading process is triggered by a randomly selected 1% of infectious individuals. After the disease breaks out, each susceptible individual *i* obtain infection with probability $1-{\left(1-\beta \right)}^{A_{\inf(t)}}$, where *A*_*inf(t)*_ is the contact number of *i* with infected individuals (effective contact number) at time *t*. Once an individual is infected, he will recover from the infection and become susceptible at a rate of γ at each time point. All simulations are processed on a scale-free network of 2000 nodes with degree distribution *p*(*k*) ∼ *k*^−2.35^ (for most real social networks, the exponent is between 2 and 3). The dynamic simulation process is terminated when there is no infected node in the network or when the simulation time is over 1000 steps. The following results of Fig 1 and Fig 2 are the average of 50 independent realizations, results of Figs 3–7 are the average of 30 independent realizations.

### The effect of contact patterns on the epidemic dynamics

In this part, we investigate the effect of the contact patterns on the epidemic dynamics without considering the influence of information on the contact behavior of individuals. We denote the number of final infected nodes in the network as I^∞^. A larger I^∞^ means heavier spreading. Fig 1 illustrates how the initial contact number *A*_*0*_ affects the epidemic prevalence and epidemic transmission threshold by increasing the effective transmission rate λ under different ratios of acquaintance contacts. Fig 1 shows that the contact number *A*_*0*_ has a similar effect on the final epidemic size and epidemic transmission threshold under different ratios of acquaintance contacts. The epidemic transmission threshold decreases with *A*_*0*_; namely, the epidemic can spread through the population more easily as *A*_*0*_ increases. When the effective transmission rate *λ* is not very big, the final epidemic prevalence increases with *A*_*0*_. However, when *λ* is large enough, for example *λ* = 1, the epidemic can spread easily through the entire population. In this situation, the effect of *A*_*0*_ is not so evident, and the final epidemic size is nearly the same. The effect of contact number *A*_*0*_ on disease spreading dynamics is consistent with the conclusion of [11].

Fig 2 illustrates how the ratio of acquaintance contacts influences the disease transmission dynamics under different contact numbers *A*_*0*_. From Fig 2, the proportion of acquaintance contacts *μ* also has an effect on the final epidemic prevalence and epidemic threshold. When the contact number is fixed, the epidemic transmission threshold will increase as *μ* goes beyond a certain value under different contact numbers. In most cases, greater *μ* leads to a smaller epidemic prevalence. At the same time, from Figs 1 and 2, we can see that reducing contact number can increase epidemic transmission threshold more effectively than increasing the proportion of acquaintance contacts. Therefore, when the epidemic outbreaks in the population, we should first reduce exposure to the disease as much as possible, and it is wise to confine our activities to social or spatial neighbors.

### Protective strategies

In this part, we study the effects of three strategies on the spread dynamics of disease. We mainly compare the effective of strategy 2 and strategy 3 and take strategy 1 as a baseline. We compare strategy 2 and strategy 3 under three situations: *a* = 0.2, *a* = 0.5 and *a* = 1. To focus on the effect of three strategies, we fix other parameters as *A*_*0*_ = 5, *μ* = 0.6, *b = 0*.*2*, *c = 0*.*2*, and *δ = 0*.*5*. Under these three parameter combinations, we study how the epidemic threshold and final prevalence varies with the effective transmission rate *λ* under different strategies. The results show that strategy 2 and strategy3 are significantly superior to strategy 1 and strategy 1 has the smallest epidemic transmission threshold. In most cases, strategy 3 yields a smaller final prevalence than strategy 2 when strategy 2 and strategy 3 have the same epidemic transmission threshold (Fig 3-1 and 3-3). Even in the case of *a* = 0.5, the epidemic threshold of strategy 3 is bigger than strategy 2 (Fig 3-2). The results imply that the difference between strategy 2 and 3 depends the impact of information. When the impact of information is very small (*a* = 0.2), the adjustment of contact behavior is unobvious, then the difference between strategy 2 and strategy 3 is not large. When the impact of information is very larger (*a* = 1), strategy 2 and strategy 3 can both enhance the epidemic transmission threshold, we can only observe the difference between strategy 2 and strategy 3 from the final epidemic prevalence. When the impact of information is medium (*a* = 0.5), the difference between strategy 2 and strategy 3 is obvious, strategy 3 can enhance the epidemic transmission threshold, strategy 2 can reduce the final propagation range but cannot change the epidemic transmission threshold. The above result implies that adjusting contact number is an effective strategy to control the spread of disease, as shown in previous studies [11]. However, adjusting the contact pattern can improve the effectiveness of adjusting the contact number. Therefore, in addition to taking some compulsory measures (closing schools, canceling some flights, etc.), governments can also use education to influence contact behavior of people and help people take effective self-protective measures. For example, when disease occurs, governments can release disease-related information through the mass media, thus enhancing the protective awareness of people and educating them to adopt effective and feasible measures; this may include canceling some public activities (community activities), reducing the use of public transport (for example, by biking or walking if traveling to a nearby location) and avoiding crowds. These self-protective behaviors could eventually achieve the goal of eliminating the disease.

### The effect of the information influences on the epidemic dynamic

In this part, we study the effect of three information influences on the epidemic dynamics. The influencing factors of contact information, local information and global information are *a*, *b*, and *c*, respectively. With more influencing factor of information, the impact of this information on individual contact behavior is also greater. From Fig 4, we can see that as *a* increases, the final epidemic size decreases sharply, and the epidemic size becomes 0 beyond a certain value. In addition, we can obviously see that strategy 3 can better prevent the spread of disease than strategy 2. The impact of local information on the epidemic dynamics is also related to the attenuation coefficient *δ*. Thus, we explored the impact of local information and attenuation coefficient *δ* together on the epidemic dynamics. From Figs 5 and 6 (corresponding the effect of local information on disease transmission dynamics in the cases of strategy 2 and strategy 3, respectively), we find that the final epidemic size decreases with *b* (local information). In addition, when the influencing factor of local information b is not very large, the decreasing tendency for different attenuation coefficient *δ* is nearly the same. But as the influencing factor of local information b increases to a large value, we can observe that the largest attenuation coefficient *δ* means the largest final prevalence. In other words, when individuals’ contact behavior is affected only by direct neighbors, the final range is largest. Namely, the epidemic is more likely to be controlled when individuals take more information into consideration.

Fig 7 shows the relationship between *c* (global information) and the final epidemic size. We conclude that the final epidemic size decreases as *c* increases. In summary, contact information has the greatest impact. Local information and global information have a modest influence, and the influence of local information on the prevalence of disease is affected by the attenuation coefficient *δ*.

## Conclusions

Contact patterns are important factors that affect the dynamic of disease transmission. A good understanding of the effect of contact patterns on the epidemic dynamics could be exploited to control the spread of disease by imposing some effective influence on the contact patterns of people. Due to the complexity of the contact behavior of people, the simple assumptions of a fully mixing population and fixed contact network structure cannot capture the real contact behavior of the population. Therefore, taking both contacts among acquaintances (network structure) and contacts among strangers (fully mixed) into consideration is more reasonable. Based on previous studies, we classified contact into acquaintance contacts and stranger contacts and explored the effect of different contact patterns on the disease dynamics. We further investigate the effects of contact behavior adjustment based on three types of disease related information. Different from previous studies, we used the infection proportion of surrounding neighbors within three degrees of separation as local information. In addition, we proposed three strategies when adding stranger contacts into the contact behavior of people and compared the effectiveness of the three strategies.

The simulation results show that the threshold of infectious disease is not only affected by the contact frequency but also influenced by the ratio of acquaintance contacts to stranger contacts. The greater the ratio, the more difficulty the disease has in spreading through the population. However, adjusting the ratio of acquaintance contacts and stranger contacts is not as effective as adjusting the contact frequency. Moreover, first reducing stranger contacts may better inhibit disease spreading. A possible explanation is that reducing stranger contacts could confine disease to a local region and contribute to the reduction of disease. In addition, epidemics can be better controlled when the attenuation coefficient of influence decreases; this means that epidemics can be best controlled when neighbors with three degrees of separation have identical influence on contact behavior.

In this study, we assumed that each node has identical initial contact frequency and the same ratio of acquaintance contacts to stranger contacts. However, heterogeneity exists in contact behavior, which means that different nodes have different contact frequencies and different contact patterns. In the future, we can study the effect of heterogeneity of contact patterns on epidemic dynamics. In addition, there are some hub nodes in scale-free networks, and these hub nodes play an important role in disease transmission. We can further study the role of hub nodes in epidemic spread with different contact patterns.
